# The Effect of Tobacco Smoking Differs across Indices of DNA Methylation-Based Aging in an African American Sample: DNA Methylation-Based Indices of Smoking Capture These Effects

**DOI:** 10.3390/genes11030311

**Published:** 2020-03-14

**Authors:** Man-Kit Lei, Frederick X. Gibbons, Ronald L. Simons, Robert A. Philibert, Steven R. H. Beach

**Affiliations:** 1 Department of Sociology, University of Georgia, Athens, GA 30602, USA; karlo@uga.edu (M.-K.L.); rsimons@uga.edu (R.L.S.); 2 Department of Psychological Sciences, University of Connecticut, Storrs, CT 06269, USA; rick.gibbons@uconn.edu; 3 Department of Psychiatry, University of Iowa, Iowa, IA 52242, USA; robert-philibert@uiowa.edu; 4 Behavioral Diagnostics, Coralville, Iowa, IA 52241, USA; 5 Department of Psychology, University of Georgia, Athens, GA 30602, USA; 6 Center for Family Research, University of Georgia, Athens, GA 30602, USA

**Keywords:** smoking, DNA methylation-based aging, African American, self-report cigarette consumption, methylation-based assessment

## Abstract

Smoking is one of the leading preventable causes of morbidity and mortality worldwide, prompting interest in its association with DNA methylation-based measures of biological aging. Considerable progress has been made in developing DNA methylation-based measures that correspond to self-reported smoking status. In addition, assessment of DNA methylation-based aging has been expanded to better capture individual differences in risk for morbidity and mortality. Untested to date, however, is whether smoking is similarly related to older and newer indices of DNA methylation-based aging, and whether DNA methylation-based indices of smoking can be used in lieu of self-reported smoking to examine effects on DNA methylation-based aging measures. In the current investigation we examine mediation of the impact of self-reported cigarette consumption on accelerated, intrinsic DNA methylation-based aging using indices designed to predict chronological aging, phenotypic aging, and mortality risk, as well as a newly developed DNA methylation-based measure of telomere length. Using a sample of 500 African American middle aged smokers and non-smokers, we found that a) self-reported cigarette consumption was associated with accelerated intrinsic DNA methylation-based aging on some but not all DNA methylation-based aging indices, b) for those aging outcomes associated with self-reported cigarette consumption, DNA methylation-based indicators of smoking typically accounted for greater variance than did self-reported cigarette consumption, and c) self-reported cigarette consumption effects on DNA methylation-based aging indices typically were fully mediated by DNA methylation-based indicators of smoking (e.g., PACKYRS from GrimAge; or cg05575921 CpG site). Results suggest that when DNA methylation-based indices of smoking are substituted for self-reported assessments of smoking, they will typically fully reflect the varied impact of cigarette smoking on intrinsic, accelerated DNA methylation-based aging.

## 1. Introduction

Smoking is a leading preventable cause of mortality and morbidity in the United States, with over 400,000 Americans dying prematurely from tobacco consumption each year [1]. The negative effect of smoking appears to be particularly pronounced among African Americans, with the Centers for Disease Control and Prevention (CDC) reporting that tobacco use is a major contributor to each of the four leading causes of death among African Americans—heart disease, cancer, and stroke and diabetes. The risk of developing diabetes is 30–40% higher for African American cigarette smokers than for nonsmokers. Likewise, African Americans, particularly males, experience lung cancer at higher rates than Whites, with the estimated smoking-attributable deaths 22% greater for African Americans [2]. Accordingly, despite smoking fewer cigarettes overall, and beginning to smoke cigarettes at an older age, African Americans are more likely to die from smoking-related diseases than Whites [3,4,5,6], and to experience greater smoking-related morbidity. This background suggests the value of focusing on examining cigarette smoking effects among African Americans.

Recent rapid progress in the development of DNA methylation-based assessment of smoking, as well as DNA methylation-based assessment of accelerated chronological aging, morbidity risk, and mortality risk, suggests the potential to examine smoking’s effect on morbidity and mortality by examining its association with DNA methylation-based age acceleration indices. Because of the potential for differential effects across ethnic groups, examining associations among African Americans is particularly important to develop tools that can provide individualized feedback to African American smokers regarding health risks and smoking effects on increased risk for morbidity and mortality. In addition, if DNA methylation markers of smoking could be substituted for self-reported smoking, this would expand the number of data sets that could be used to explore the link between smoking and pathways to accelerated morbidity and mortality among African Americans, and facilitate examination of high-risk groups who may be less likely to report smoking accurately [7]. Demonstration of the utility of DNA methylation-based indicators of smoking in fully mediating the effect of self-reported assessment of smoking on indices of accelerated DNA methylation-based aging would be a step forward for both ongoing research related to African American health and for rapid translation to clinical practice [8,9,10].

There has been substantial progress in the use of DNA methylation to quantify exposure to cigarette consumption. This began with early work identifying smoking associated changes in level of methylation of the CpG residue referred to as cg05575921 in the aryl hydrocarbon receptor repressor (AHRR) [11,12,13,14,15,16,17,18], and work has expanded recently to provide a useful comprehensive index of “pack-years” (PACKYRS) of cigarette consumption [19] that provides estimated total pack-years of cigarette consumption. These tools provide the opportunity to examine the link between smoking and a range of DNA methylation-based measures, especially in contexts in which self-reported measures of cigarette consumption may be unreliable [20,21,22]. In addition, use of epigenetic biomarkers of substance use may allow feedback and advice without requiring patients to report their smoking status, and may also better capture low-level or indirect exposures that may be more common for African Americans [23].

Several studies have shown that smoking is associated with accelerated DNA methylation-based aging [8,24,25], as well as with epigenome-wide DNA methylation in whole blood [26], and also with DNA methylation-based predictors of increased mortality risk [27,28]. Likewise, smoking cessation has been reported to lead to reductions in DNA methylation-based aging [10], suggesting that quitting smoking may lead to epigenetic alterations. Recently, DNA methylation-based PhenoAge [19], estimated using 513 CpG sites, has been developed to predict morbidity and health span, whereas DNA methylation-based GrimAge [29], estimated using 1030 CpG sites, was developed to be an improved predictor of all cause mortality. However, prior work has also found some inconsistent effects of smoking on accelerated aging, with Levine et al. (2018) [29] reporting little effect of pack years of smoking on PhenoAge, whereas Yang et al. (2019) [25] found an effect of self-reported packyears on phenotypic aging and noted it was strongest for current smokers. These findings suggest the possibility that current smoking may be more strongly associated with DNA methylation-based aging indices than is history of past consumption. In addition, Zhao et al. (2019) [30] found that self-reported smoking was related to accelerated aging for both accelerated PhenoAge and for accelerated GrimAge, whereas Horvath and Haj (2018) [31] report that smoking-related methylation changes did not influence the Horvath [32] or Hannum et al. [33] clocks, but was related to “PhenoAge” [29]. These findings suggest that the association of self-reported smoking or DNA methylation-based indices of smoking may differ across different indices of accelerated aging.

Given inconsistencies in the literature, there are several dimensions of difference between widely used measures of DNA methylation-based aging that should be noted. First, there are differences in what they are designed to predict, i.e., whether they reflect chronological age, morbidity, or mortality risk Second, there are differences due to whether they correct for chronological age to yield an age acceleration measure. And, third, there are differences introduced by whether they incorporate controls for likely individual differences in the distribution of underlying heterogenous cell-types. Comparing different indices using different control variables may make comparisons more difficult to interpret. Accordingly, below we review the different DNA methylation-based indices to be used in the current investigation, noting that in each case, our primary analyses will focus on examination of an age acceleration index (i.e., controlling for chronological age) and will control for individual differences in cell-type distribution (i.e. to generate an “intrinsic” DNA methylation-based aging measure). Sensitivity analyses will examine associations without controls for cell-type variation (i.e. “extrinsic” aging measures). In line with prior literature, we expect that smoking, and DNA methylation-based indicators of smoking, will be more strongly related to measures of accelerated phenotypic aging that reflect morbidity, or to measures of accelerated aging designed to capture increased mortality risk, than it is to DNA methylation-based measures designed to reflect accelerated chronological aging.

Given their differences, it is important to review the DNA methylation-based aging indices used in the current investigation. Briefly, we examine DNA methylation-based measures of (a) accelerated chronological age, (b) accelerated phenotypic health age, (c) accelerated mortality risk age, and (d) telomere length. We do not examine the full range of possible DNA methylation-based aging measures which have continued to expand (see Bell et al., 2019 for a review) [34], but rather examine the most widely used exemplars. Likewise, we do not examine the full set of DNA methylation-based indices of smoking, which have also expanded substantially, choosing instead to focus on an early simple, single locus index (cg05575921) and a more recent complex index that is widely available (PACKYRS). Adding to their potential attractiveness to researchers, all the indices examined can be assessed using a single, widely available DNA methylation platform. For each of the DNA methylation-based aging measures reviewed below, the residual, or error relative to chronological age, can be used to index biological age acceleration and is typically seen as superior to the uncorrected measure in predicting health outcomes for those of heterogeneous ages.

### 1.1. Chronological Aging

Early epigenetic indices used chronological age as the criterion variable to be predicted. Pioneering work by Horvath focused on the way in which DNA methylation patterns across tissues followed a regular pattern of change reflecting chronological age (see Horvath, 2013 [32]; Horvath & Rak, 2018 [31]). This allowed Horvath and colleges to identify an optimal set of DNA methylation-based predictors of chronological age. Using a similar approach, but focused on peripheral blood only, Hannum and colleagues devised an additional DNA methylation-based index focused on prediction of chronological age, using a largely non-overlapping set of weighted methylation values [33]. These initial measures of DNA methylation-based aging, focused on chronological age, were designed to have a relatively constant rate of change across adulthood after age 20 and had a high correlation with chronological age [35]. As with the other DNA methylation-based measures of aging reviewed below, these initial chronological aging measures were built using a supervised machine learning method to identify an informative set of CpGs [29,36].

### 1.2. Phenotypic (Morbidity) Aging

DNA methylation-based indices have also been developed to predict disease phenotypes in combination with chronological age. In particular, the “PhenoAge” DNA methylation index (DNAm PhenoAge) developed by Levine et al. (2018) is currently in widespread use [29]. The DNAm PhenoAge index was designed to overcome some limitations of the first generation of chronological measures [32,33]. Specifically, acceleration of the chronological aging indices was not found to be consistently related to cardiovascular disease or early onset of chronic illness [37]. In response, DNAm PhenoAge was developed [29,31] using both chronological age and clinical measures so that it would better predict individual differences in lifespan and healthspan. The index reflects several known aging pathways, including the Janus kinase-signal transducer and activator of transcription (JAK-STAT) cascade, the response to lipopolysaccharide, and the tumor necrosis factor-mediated signaling pathway [29,31], and provides a useful objective marker of elevated risk for early onset morbidity and chronic illness.

### 1.3. GRIM and Its Subscales

Increased morbidity does not always translate into increased risk for mortality. To address this potential limitation, Lu and colleagues recently developed a DNAm-based measure of predicted lifespan, focusing on the prediction of time to death due to all-cause mortality [19]. They developed the measure by identifying a set of plasma protein predictors of mortality and then using these protein predictors to identify DNAm-based biomarkers that could predict mortality. The resulting index allows accurate prediction of time-to-death, providing a mortality risk estimate called “DNAm GrimAge.” The index has demonstrated good predictive ability for time-to-death, time-to-coronary heart disease, time-to-cancer, and has also shown an association with computed tomography data for fatty lever/excess visceral fat, and age at menopause [19].

Because the GRIM scale was designed to include DNA methylation-based indicators of smoking, DNA methylation-based indicators of smoking will show associations with GRIM due to part-whole correlations, and this also affects interpretation of association of self-reported smoking with GRIM. To provide an alternative assessment of connections between smoking and GRIM, we also examine associations with each of the seven subscales of GRIM, i.e., other than DNA methylation-based pack-years (PACKYRS), which were designed to capture specific protein predictors of increased mortality risk and so are not confounded with pack-years assessment: Adrenomedullin (ADM), beta-2 microglobulin (B2M), growth differentiation factor 15 (GDF15), Cystatin C (CystatinC), leptin (Leptin), plasminogen activation inhibitor 1 (PAI1), and tissue inhibitor metalloproteinase 1 (TIMP1).

### 1.4. Telomere Length

Lu e al. (2019) developed a DNA methylation-based index of telomere length (mTL), i.e., the number of repetitive nucleotide sequences at the end of each chromosome that shortens with increasing number of replications [38]. Correlations between telomere length (TL) and age range between r = −0.513 for women and r = −0.552 for men [39]. Telomere length has long been of interest with regard to aging, but it has been considered problematic by some researchers due to difficulties in obtaining reliable measurement [40]. The DNA methylation-based index developed by Lu and colleagues (2019) [38] attempts to address this problem. Although it was developed to predict criterion measures of TL, mTL demonstrated superior prediction of health outcomes as well as stronger associations with health behaviors including smoking. A cautionary note offered by Lu et al. (2019) is that mTL may reflect number of replications of each cell has undergone more faithfully than it reflects actual TL, so it should not be assumed that mTL will faithfully reflect directly measured TL. 

### 1.5. Controlling Cell-type Variation

For each of the measures described above it is possible to use the measure as originally derived, or alternatively, to control for individual differences in cell type variation to generate an “intrinsic” measure that is less influenced by individual differences in composition of blood across individuals. Various methods have been described in the literature by which DNA methylation patterns can be used to identify and correct for individual differences in particular cell-types. Because these individual differences may account for, or confound, associations with morbidity and mortality risk, and because they may have somewhat different impact on different aging indices, it may be useful to control for cell-type variation when comparing effects across different indices. Conversely, it should be noted that some of the association between smoking and health outcomes could be the result of effects of smoking on cell-type variation [41,42]. However, prior research with African Americans has not found that correcting for cell-type variation decreases the association of lifestyle factors such as smoking with accelerated aging indices of morbidity or mortality [30]. In the current investigation, therefore, for ease of comparison across indices, we present primary analyses controlling for cell type using the procedure described by Horvath [32]. However, we also provide results uncorrected for individual variation in cell-type in the sensitivity analysis.

As outlined above, the development of reliable DNA methylation-based indicators of smoking along with the development of robust measures of cellular level aging, phenotypic aging, and elevated mortality risk, provides an opportunity to better understand the impact of smoking on health and to provide better feedback regarding biological impact. However, an important question remains in the examination of the utility of DNA methylation-based indicators of smoking as an alternative to self-reported smoking. Do DNA methylation-based indicators of smoking fully account for the impact of self-reported smoking assessment of smoking status? To the extent that DNA methylation-based indicators of smoking fully mediate the association between self-reported smoking and DNA methylation-based indices of morbidity and mortality, it may be reasonable to use them in place of self-report assessment to capture smoking’s effects on biological aging and increased mortality risks. Accordingly, in the current investigation we examine the possibility that DNA methylation-based indicators of smoking may fully mediate the impact of self-reported measure of smoking on intrinsic measures of accelerated DNA methylation-based health indictors. 

### 1.6. Specific Hypotheses

**Figure 1:**
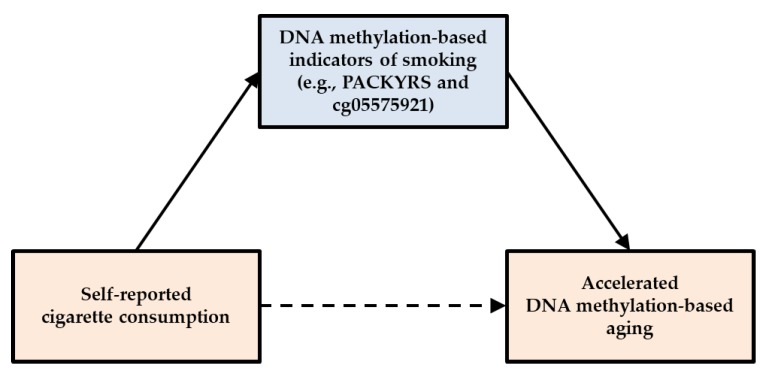
Theoretical model linking self-reported cigarette consumption to accelerated aging outcomes through DNA methylation-based indicators of smoking.

Figure 1 shows the theoretical model tested in the current study. We hypothesized a) that self-reported cigarette consumption would be associated with DNA methylation-based aging indices indicating a deleterious effect on cellular level aging across a range of measures of accelerated aging, possibly with stronger associations for measures specifically designed to reflect morbidity and increased mortality risk, b) that predictive effects would be stronger for DNA methylation-based indicators of smoking (e.g., cg05575921 or PACKYRS) than for self-reported smoking, and c) that DNA methylation-based indicators of smoking would fully mediate the effect of self-report measures of smoking on accelerated cellular aging.

## 2. Materials and Methods 

### 2.1. Sample

We tested hypotheses using data from the Family and Community Health Study (FACHS). FACHS is a longitudinal study of 889 African American families (children and their primary and secondary caregivers) that was initiated in 1997. All the families had a 5th grader at study inception. Using a stratified random sampling procedure, the sampling strategy was intentionally designed to generate families representing a range of socioeconomic statuses and neighborhood settings. Details regarding recruitment are described by Gibbons and colleagues (2004) and Simons and colleagues (2011) [43,44]. At Wave 1, about half of the sample resided in Georgia (*n* = 422) and the other half in Iowa (*n* = 467). The 4th and 5th Waves of data were collected in 2005 and 2008, respectively. At wave 5, 17.82% of primary caregivers had less than a 12th grade education, and 24.8% were married. The majority (68.5%) lived in large urban areas, 12.2% lived in the suburbs, and 19.3% lived in rural areas. 

Of the caregivers interviewed at Wave 1, 77% were interviewed again at Wave 5. Within two weeks of the wave 5 psychosocial interview, a certified phlebotomist visited the home and collected four tubes of blood (30 mL) from each consenting participant. Given the logistics of scheduling home visits by phlebotomists, only members of the sample still residing in Georgia or Iowa at wave 5 were identified as eligible for the blood draw. Roughly 80% of these individuals agreed to provide blood: 377 women and 129 men. Comparisons of these samples with those who did not provide blood did not reveal any significant differences with regard to demographic characteristics at the first wave of data. After eliminating missing cases, complete data were available for 500 middle-age African Americans (375 women and 125 men). Rates of missing data ranged from 0.40% for BMI to 0.79% for cigarette use.

All study protocols and procedures were approved by the Institutional Review Board at the University of Georgia (Title: FACHS weathering - Targets, Study approval number 00006152).

### 2.2. Procedures

The questionnaires were administered in the respondent’s home and took on average of about two hours to complete. The instruments were presented on laptop computers. Questions appeared in sequence on the screen, which both the researcher and participant could see. In an effort to further enhance anonymity, the questionnaires were administered using audio-enhanced, computer-assisted, self-administered interviews (ACASI). Using this procedure, the respondent sat in front of a computer and responded to questions as they were both presented visually on the screen and auditorily via earphones.

In addition, participants were also asked to provide a blood sample at Wave 5. The phlebotomist drew four tubes of blood (30 mL) from each participant; these were shipped on the same day to a laboratory at the University of Iowa for preparation. Whole blood DNA was prepared using cold protein precipitation, quantified with a NanoDrop photometer (Thermofisher, 168 Third Avenue Waltham, MA, USA) and stored at −20 °C until use [45].

### 2.3. DNA Methylation Procedures

For the current study, FACHS subjects were assessed for genome-wide methylation status using our standard protocols [18,46]. Upon receipt, the blood tubes were inspected to ensure anticoagulation and aliquots of blood were diluted 1:1 with phosphate buffered saline (pH 8.0). Mononuclear cell pellets were separated from the diluted blood specimen using a centrifuge with ficoll (400 ×g, 30 min). The mononuclear cell layer was removed from the tube using a transfer pipette, re-suspended in a phosphate buffered saline solution, and briefly centrifuged again. The resulting cell pellet was re-suspended in a 10% DMSO/RPMI solution and frozen at 8.0 °C until use. Genomic DNA was prepared from ficoll purified peripheral mononuclear cell DNA pellets using a Qiagen (19300 Germantown Road, Germantown, MD, USA) DNA Mini Kit according to manufacturer’s directions. A typical DNA yield for each mononuclear cell pellet was between 10 and 15 mg. 

DNA methylation-based assessments were conducted with the Illumina Infinium (Sequenom, Inc., San Diego, CA, USA) HumanMethylationEPIC 850 BeadChip. This array contains 865,918 probes recognizing CpG positions of known transcripts, potential transcripts or CpG islands. Participants were randomly assigned to 16 sample “slides/chips” with groups of eight slides being bisulfite converted in a single plate, resulting in two “batches/plates.” A replicated sample of DNA was included in each plate to aid in assessment of batch variation and to ensure correct handling of specimens. The replicate sample was examined for average correlation of beta values between plates and was found to be greater than 0.99. Prior to normalization, DNA methylation data were filtered based on these criteria: (a) samples were examined to identify any “poor quality samples” containing 1% or more of CpG sites with detection *p* < 0.05 (but, no samples were found to fail this criterion), (b) sites were removed if a bead count of < 3 was present in 5% of samples, and (c) sites with a detection *p* < 0.05 in 1% of samples were removed. 

The beta value at each CpG locus was calculated as the ratio of the intensity of the methylated probe to the sum of intensities of the methylated and unmethylated probes. Quantile normalization methods were used, with separate normalization of Type I and Type II assays, as this approach has been found to produce marked improvement for the Illumina array in detection of relationships by correcting distributional problems inherent in the manufacturers default method for calculating the beta value. Finally, beta values after quantile normalization were used to calculate DNA methylation-based aging indices and DNA methylation-based telomere length for each participant using a public online tool (https://dnamage.genetics.ucla.edu/).

### 2.4. Measures

#### 2.4.1. Self-reported Cigarette Consumption 

Self-reported cigarette consumption at three waves of data collection were used to establish smoking history across an 8-year period (from waves 3 to 5). At each wave of data collection, subjects were asked, “In the past month, how much did you smoke cigarettes?” Response options included 0 = none at all, 1 = less than 1 cigarette a day, 2 = 1–5 cigarettes a day, 3 = about half a pack a day, 4 = about a pack a day, 5 = about 1 and a half packs a day, and 6 = about 2 packs a day. Pearson’s correlations across three waves of data collection were above 0.78. Thus, scores were averaged across the three waves of data collection to calculate average cigarette consumption across the 8-year period ($\bar{x}$ = 0.888, *SD* = 1.296).

#### 2.4.2. Accelerated DNA Methylation-Based Aging

We assessed DNA methylation-based aging using established procedures to calculate each of the previously established DNA methylation-based clocks including the Hannum index [33], the Horvath index [32], the PhenoAge index [29], and the GrimAge index [19]. All indices were analyzed using the online “New Methylation Age Calculator” (https://dnamage.genetics.ucla.edu/) with the Advanced Analysis option and the normalize data option. First, the Hannum index is computed using 71 CpG sites scattered throughout the human genome where DNA methylation levels correlate strongly with chronological age. Second, the Horvath method utilizes information from 353 CpG sites. Third, a DNA methylation-based measure of accelerated phenotypic aging (PhenoAge) was developed using both age and clinical measures so that it would better predict individual differences in lifespan and health span. The index is based upon 513 CpG sites that reflect several known aging pathways [31]. Finally, the GrimAge index estimates an individual’s biological age based on DNA methylation assessments at 1030 sites scattered across the human genome. 

#### 2.4.3. DNA Methylation-Based Estimate of Telomere Length 

Lu and colleagues (2019) identified 140 CpG sites to estimate telomere length in Kb. Similar to accelerated aging [38], adjusting DNA methylation-based telomere length (mTLadjAge) was measured using the residual from regressing DNA methylation-based telomere length (mTL) on chronological age. A positive value would indicate mTL that is longer than expected based on chronological age. We used the (https://dnamage.genetics.ucla.edu/) website to calculate the mTL index [38].

#### 2.4.4. Components of GrimAge 

GrimAge components were developed using DNAm indicators of eight individual predictors of mortality, including seven plasma proteins: adrenomedullin (ADM), beta-2 microglobulin (B2M), growth differentiation factor 15 (GDF15), Cystatin C (CystatinC), leptin (Leptin), plasminogen activation inhibitor 1 (PAI1), and tissue inhibitor metalloproteinase 1 (TIMP1). In addition, GrimAge also included the variable “smoking pack-years” (PACKYRS) based on DNAm indicators. The DNA methylation-based indicators were combined to create the GRIM index. Individual subscale scores are also available at the (https://dnamage.genetics.ucla.edu/) website, providing our measures of adrenomedullin (ADM), beta-2 microglobulin (B2M), growth differentiation factor 15 (GDF15), Cystatin C (CystatinC), leptin (Leptin), plasminogen activation inhibitor 1 (PAI1), and tissue inhibitor metalloproteinase 1 (TIMP1) as well as “smoking pack-years” (PACKYRS). 

#### 2.4.5. Transformation to Accelerated Aging 

To transform each DNA methylation-based age into an accelerated aging score, we formulated a measure of accelerated aging using the unstandardized residual scores from the regression of DNA methylation-based age on chronological age [47]. These residuals had a mean of zero and represented both positive and negative deviations from chronological age (in years), with positive scores indicating accelerated aging.

#### 2.4.6. Cell-Type Composition 

To adjust for cellular heterogeneity that can influence DNA methylation-based values, we controlled for cell-type distribution in the models. Cell-type composition was estimated using the “EstimateCellCounts” function in the “minfi” Bioconductor package, which is based on the reference-based method developed by Houseman and colleagues [48]. Using this approach, we estimated cell-type proportions in whole blood for CD4+ T cells, CD8+ T cells, Natural Killer cells, B cells, and monocytes. These cell-type proportions were controlled for to examine associations between DNA methylation-based aging measures and predictors that were relatively free of potentially confounding cell-type variation influences. In this way the associations reflect “intrinsic” accelerated aging measures that are relatively independent of cell-type differences between individuals.

#### 2.4.7. Control Variables 

To account for variables that could provide plausible rival explanations, we controlled for gender (1 = males) and body mass index [BMI = weight in kilograms/(height in meters)^2^] as these have been correlated with accelerated DNA methylation-based aging in prior research [19,29,38].

### 2.5. Analytic Approach

We first examined partial correlation coefficients between DNA methylation-based age indices and smoking-related variables (e.g., self-reported cigarette consumption, DNA methylation-based smoking consumption, and cg05575021), controlling for BMI, gender, and cell-type composition. When the partial correlation coefficients were significant, we then examined regression models in which self-reported cigarette consumption and all controls were examined in Step 1, DNA methylation-based smoking consumption was added in Step 2, and cg05575921 was added in Step 3. Finally, we follow the common usage as described by Hayes [49]. The direct effect is the association between self-reported cigarette consumption and the outcome that is left after including DNA methylation-based indicator of smoking in the analysis. In contrast, the indirect effect is the effect of self-reported cigarette consumption on the outcome through the DNA methylation-based indicator of smoking. When the confidence interval for the “direct effect” includes zero, it indicates that the previously significant association has been reduced to non-significance. Thus, to assess the significance of direct and indirect effects, 95% confidence intervals (CI) were estimated with bias-corrected and accelerated bootstrapping with 1000 resamples using Mplus software (Version 8) [50].

## 3. Results

### 3.1. Initial Findings

Average age was 48.791 years (*SD* = 8.345). The age range (26.92 to 92.17) for the subjects in the studies extends from early adulthood to retirement age. Average cigarette consumption in the sample over eight years was 0.888 (*SD* = 1.296), i.e., less than 1 cigarette a day. For the 41 percent of participants who smoked during waves 3 to 5, average consumption was 2.176 (*SD* = 1.145) per smoker, i.e. between a few cigarettes a day and a half a pack a day. As shown in Table S1, gender, BMI, and cell-type composition indices were associated with cigarette use and with DNA methylation-based aging indices (See Table S1). Table 1 presents partial correlations for all study variables, controlling for gender, BMI, and cell-type composition (See Table S2 for Pearson’s correlation coefficients).

There was no significant partial correlation between first-generation DNA methylation-based aging (Hannum and Horvath’s methods) and smoking-related variables (e.g., cigarette consumption, DNA methylation-based smoking consumption, and cg05575021). In contrast, there were significant partial correlations between new-generation DNA methylation-based aging (e.g., PhenoAge, mTL, and Grim) and all smoking-related variables.

### 3.2. Model Testing

As can be seen in Table 1, three of the five measures of intrinsic age acceleration were associated significantly with self-reported cigarette consumption as well as with smoking assessed by the pack-year subscale of the GRIM (PACKYRS) and DNA methylation level of the CpG site cg05575921 in intron 3 of the aryl hydrocarbon receptor repressor (AHRR) gene. In addition, two of the seven subscales of the GRIM, excluding PACKYRS, were also associated with all three indicators of smoking. Measures of intrinsic aging that did not show this pattern were not associated with any of the indicators of smoking. However, one of the subscales of GRIM (CystatinC) was associated with both self-reported smoking and PACKYRS but not cg05575021. Table 2 shows the results of using OLS regression analyses to examine the potential indirect effects for scales showing an association between accelerated DNA methylation-based aging and smoking. Using PhenoAge as the dependent variable, Model 1 shows that the significant main effect of self-reported cigarette consumption (β = 0.111, *p* = 0.007) on PhenoAge was reduced to non-significance when either PACKYRS (Model 1A) or cg05575921 (Model 1B) was added to the set of predictors. This pattern did not indicate full mediation of smoking’s effect because the DNA methylation-based indicators of smoking were not significant predictors in these regressions. 

Shifting the focus to DNA methylation-based assessment of telomere length as the outcome, Model 2 shows a significant main effect of self-reported cigarette consumption (β = −0.231, *p* < 0.001), suggesting that shorter telomere length was observed in smoking individuals. Further, Models 2A and 2B added PACKYRS and cg05575921, respectively. Consistent with full mediation, the effect of self-reported cigarette consumption on telomere length was no longer significant when either PACKYRS or cg05575921 was included in the model, but the hypothesized mediators, PACKYRS (β = −0.168, *p* = 0.005) and cg05575921 (β = 0.211, *p* = 0.001) were significantly associated with mTL. 

Finally, in Models 3-5, we examined GrimAge and the two components significantly associated with smoking (ADM and GDF15). Models 3 through 5 in Table 2 each show a pattern of results supportive of mediation and similar to those in Model 2. The findings indicate that there was a significant main effect of self-reported cigarette consumption on each accelerated aging index, but that the effect was no longer significant after taking into account either PACKYRS or cg05575921. In the case of GrimAge, when cg05575921 was entered as a predictor, the association of self-reported smoking was substantially reduced, but remained significant. When elements of potential mediation emerged, we proceeded with a formal test of direct and indirect effects using a bootstrapping method with 1000 replications.

### 3.3. Testing of Direct and Indirect Effects

**Figure 2:**
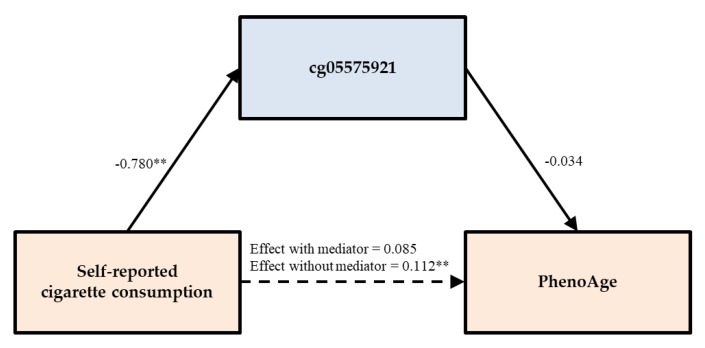
cg05575921 reduces the direct effect of self-reported cigarette consumption on PhenoAge to non-significance. Note. Chi-square = 41.539, *df* = 7, *p* = 0.000; CFI = 0.944; SRMR = 0.041. Values are standardized parameter estimates. ** *p* ≤ 0.01; * *p* ≤ 0.05 (two-tailed tests).

To further examine the indirect effects of self-reported cigarette consumption on DNA methylation-based outcomes through either PACKYRS or cg05575921, we used bootstrapping methods with 1000 replications. As can be seen in Table 3, the tests for indirect effects of self-reported cigarette consumption on PhenoAge through either PACKYRS or cg05575921 was not significant. The test of the indirect effect of self-reported cigarette consumption on mTL through PACKYRS was significant, indirect effect (IE) = −0.126, 95% CI [−0.224, −0.023], accounting for 57.534% of the total effect. Likewise, using bootstrapping methods with 1000 replications, the test of the indirect effect of self-reported cigarette consumption on mTL length through cg05575921 was significant, indirect effect (IE) = −0.166, 95% CI [−0.264, −0.065], accounting for 75.455% of the total effect. To better illustrate the way in which direct and indirect effects were related, we plotted the mediational model for mTL through cg05575921 in Figure 2. 

Using the same method to test the direct and indirect effects, the tests of the indirect effect of self-reported cigarette consumption on GrimAge through both PACKYRS and cg05575921 were significant, accounting for 96.524% and 81.978% of the total effect, respectively. The mediation was not complete when cg05575921 was used as the mediation, as indicated by a significant direct effect of self-reported smoking on GrimAge even after including cg05575921. As can be seen in the bottom half of Table 3, the effects of self-reported cigarette consumption on two components of GrimAge (ADM and GDF15) were fully mediated by both PACKYRS and cg05575921. For ADM, the indirect effects of smoking through PACKYRS and cg05575921 were 74.699% and 76.821% of the total effect, respectively. For GDF15, the indirect effects through both PACKYRS and cg05575921 accounted for 93.966% and 81.739% of the total effect, respectively.

**Figure 3:**
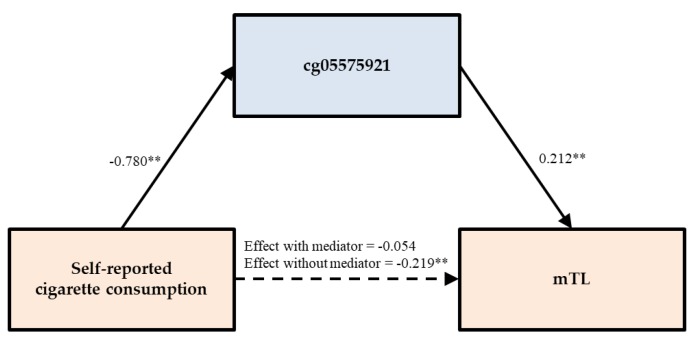
cg05575921 fully mediates the impact of self-reported cigarette consumption on mTL. Note. Chi-square = 41.539, *df* = 7, *p* = 0.000; CFI = 0.950; SRMR = 0.024. Values are standardized parameter estimates. ** *p* ≤ 0.01; * *p* ≤ 0.05 (two-tailed tests).

**Figure 4:**
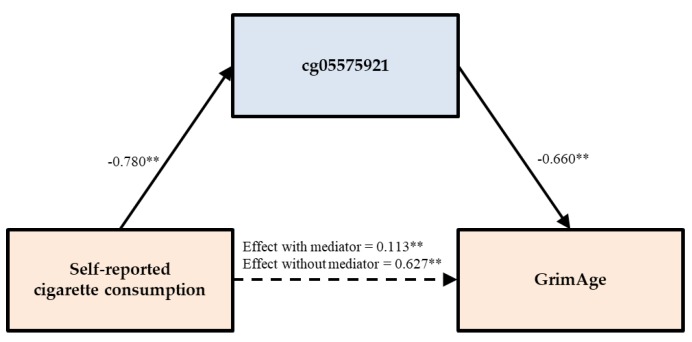
cg05575921 partially mediates the impact of self-reported cigarette consumption on GrimAge. Note. Chi-square = 41.539, *df* = 7, *p* = 0.000; CFI = 0.968; SRMR = 0.028. Values are standardized parameter estimates. **p ≤ 0.01; *p ≤ 0.05 (two-tailed tests).

To illustrate the way in which direct and indirect effects were related for mTL and for GrimAge, in Figure 3 (mTL) and Figure 4 (GrimAge) we plotted mediational models using cg05575921 as the mediator. As can be seen, the introduction of the mediator substantially reduces the direct effect, and in each case there is a significant association of self-reported with DNA methylation-based assessment of smoking, which in turn is significantly associated with the outcome, resulting in the observed significant indirect effect through the mediator.

### 3.4. Sensitivity Analysis

To examine the robustness of the results and the potential impact of cell-type variation, we then re-estimated the indirect effects for each outcome without controlling for cell-type variation. As shown in Table 4, the pattern of results is unchanged from those in Table 3, however, because previously non-significant associations became significant for PhenoAge, we were able to estimate indirect effects of self-reported cigarette consumption on PhenoAge through PACKYRS, resulting in a significant, indirect effect (IE) = 0.144, 95% CI [0.011, 0.281], accounting for 66.666% of the total effect. Finally, we repeated all analysis using only current smokers and excluding those who indicated smoking at a prior wave but no smoking at wave 5 (n = 470). Table 5 reports these results and indicates that results were largely unchanged relative to results using the full sample reported in Table 3. 

## 4. Conclusions and Discussion

The current investigation examined the impact of smoking on accelerated aging among middle-aged African Americans. Controlling for effects of gender, BMI, and cell-type compositions, there was no significant partial correlation between first-generation DNA methylation-based aging indices (i.e., Hannum et al. [33]; or Horvath [32]) and any of the smoking indices (i.e., self-reported cigarette consumption, PACKYRS, and cg05575021). The lack of association was not due to lack of variability in either accelerated aging indices or indicators of smoking. Accordingly, it is possible that DNA methylation patterns selected using chronological age as the criterion variable did not identify loci strongly influenced by smoking in this sample of middle-aged African Americans. This may not be entirely surprising as first generation epigenetic aging measures were not designed to reflect increased presence of disease phenotypes, or increased level of morbidity, or increased mortality risk. Instead, they focused on predicting individual differences in the rate of cellular level aging as reflected in epigenetic patterns found to change at a regular rate with chronological age. Because they were not focused on identifying the epigenetic effects of pathogenic environmental factors or health behaviors, unless the primary mechanism of smoking’s effect on morbidity and mortality was through increased rate of cellular level aging, it would not be influenced by smoking (or other negative health behaviors). Our hypothesis that all measures of accelerated aging would be related to cigarette smoking was therefore not confirmed.

We did, however, find evidence of an association between smoking and accelerated aging using newer measures designed to capture DNA methylation-based PhenoAge, increased mortality (Grim), and decreased telomere length or increased rate of cellular division (mTL), and these associations were robust to controls for age, gender, BMI, and cell-type compositions. The difference in patterns of effects between these newer measures designed to reflect problematic physiological changes vs. older measures designed to capture chronological age is striking. One conclusion is that measures of accelerated aging are not all providing the same information about underlying health impacts and should not be used interchangeably. Better understanding the different determinants and consequences of different accelerated aging indices may be useful in future research on public health. The associations between smoking and accelerated aging observed for the newer indices are consistent with suggestions by recent studies [19,29,38] that there may be effects of cigarette use on some DNA methylation-based aging measures and on telomere length. Also noteworthy are significant correlations of smoking indices and two subscales of the GRIM: ADM and GDF15. Both play an important role in inflammatory response.

We also examined the practical issue of whether DNA methylation-based measures of smoking would largely replicate patterns found using self-reported smoking, whether effects using DNA methylation-based measures of smoking typically would be stronger, and whether epigenetic measures would fully mediate effects of self-reported smoking. In all cases, we found that DNA methylation-based measures (both PACKYRS from GrimAge and cg05575021) performed well relative to self-reported smoking, and captured the large proportion of the effect of self-reported smoking. For those accelerated aging outcomes associated with smoking, PACKYRS from GrimAge was more strongly associated with accelerated aging than was self-reported cigarette consumption. With the exception of the PhenoAge, the association of cg05575021 with accelerated aging outcomes was also greater than that observed for self-reported cigarette consumption. Similarly, in our examination of mediation, the effect of self-reported cigarette consumption on outcomes was fully mediated by PACKYRS for Telomere length, GrimAge, ADM, and GDF, and for PhenoAge, there was no significant mediation because both self-report and the DNA methylation-based indices of smoking were reduced to non-significance when entered simultaneously. Similar results were obtained using cg05575021 as the DNA methylation-based index of smoking (PACKYRS), except that it did not fully mediate the effect of self-reported smoking on GrimAge. Accordingly, it appears the effect of self-reported cigarette consumption is largely captured by readily available DNA methylation-based indices of smoking. Sensitivity analyses examined whether the enhanced performance of DNA methylation-based indices of smoking relative to self-reported smoking could be accounted for by under-reporting among smokers (i.e., false negatives). However, partial correlation analyses removing all those with cotinine greater than 10 who reported no smoking, indicated no change in the pattern of results (see Table S3). Accordingly, it appears that DNA methylation-based measures of smoking are typically better at capturing the physiological impact of smoking and not just better at identifying smoking in research populations.

The very strong association of smoking and smoking related DNA methylation indices with GrimAge reflects, in part, the fact that a DNA methylation-based index of smoking is a component of the composite comprising GrimAge. Accordingly, the associations of smoking indices with GrimAge are inflated due to part-whole correlations. For current purposes, we note that self-reported smoking effects on Grim were fully mediated by the more comprehensive DNA methylation-based indicator of smoking (PACKYRS) and partially mediated by cg05575921 (81.978%). In addition, two of the subscales of GrimAge that were not designed to reflect smoking were also significantly associated with self-reported smoking, and these effects were also fully mediated by both DNA methylation-based indices of smoking. Also of potential interest for future research, the pattern of results was somewhat clearer when analyses were conducted without controlling cell-type variation. In the analysis presented in Table 4, all 10 direct effects examined were non-significant, whereas 9 of the 10 indirect effects were significant, demonstrating the power of DNA methylation-based indices of smoking to fully capture effects associated with self-reported smoking.

Importantly, the current results highlight the potential for existing epidemiological data sets with mDNA assessments to provide useful insights beyond those for which they were initially collected. In the area of smoking research, this potential seems considerable. In particular, studies that examined genome-wide DNA methylation but did not assess self-reported smoking can examine smoking effects using DNA methylation-based indices of smoking and obtain effects comparable to those resulting from direct self-report assessment. The current results suggest that such studies can be used to examine the impact of smoking on biological aging outcomes, and the use of DNA methylation-based indicators of smoking may allow more nuanced examination of effects on aging using larger samples. At the same time, the current results suggest caution in treating DNA methylation-based aging indices as interchangeable. The lack of similarly in the context of associations with smoking may reflect more general differences in patterns of predictors and outcomes. In particular, different patterns may emerge depending on whether chronological vs. physiologically-based indices of epigenetic aging are used. Better unraveling of the implications of these different patterns for longer-term health outcomes is an additional area of potential future investigation. We did not find substantial difference in patterns of association depending on whether we controlled for individual differences in cell-type variation, suggesting that the cell-types we controlled were not accounting for much of the association between smoking and accelerated aging. Also, patterns of association were unchanged by excluding those who reported quitting smoking at wave 5. 

Continued enhancement of public health efforts to reduce smoking requires continuing improvement in the way empirically grounded advice regarding smoking is provided to individuals who are willing to consider smoking cessation. The current findings of mediation of self-reported smoking by epigenetic indices of smoking suggest that it may be possible to provide routine feedback to individuals at their regular physical exam regarding their smoking, and the impact of their smoking on accelerated aging. It is possible that this type of feedback might prove a more compelling reason to quit for many individuals and sponsor more discussion about quitting smoking with primary care physicians.

Using widely available indices of biological aging, we found support for a hypothesized effect of smoking on accelerated aging using measures that focus on predicting physiological changes linked to health outcomes but not using measures focused on predicting chronological age only. Because we controlled for age, gender, cell-type variation, and BMI, for all indices, results are more readily comparable across indices. Interestingly, the most compelling results were obtained using a recently developed measure of mTL and a subscale of the GRIM which assesses mGDF15. If this pattern is replicated in future examinations of the association between accelerated aging and indicators of smoking, it may be useful to think of mTL and mGDF15 as sensitive, early indicators of the physiological damage accumulating due to smoking. Because they provide rather different indicators of risk for future disease both may be of considerable and independent interest. Providing feedback regarding these indicators may help motivate smokers to quit. In addition, these findings support mechanistic research into ways that smoking influences these markers and ultimately cancer, heart disease, and other illnesses leading to premature death. 

While the current study presents important findings, it has limitations that need to be noted. First, the lack of significant associations in this study may, in some cases, be due to sample size. Increased sample size would increase power to detect significant relationships even when effect sizes are small. Accordingly, a greater sample size would likely have identified some relationships as significant that were not significant in the current study and so may have identified additional direct associations of self-reported smoking with DNA methylation-based indicators of accelerated aging. Second, we did not examine individual probes in the current study. This could be important for future efforts to characterize smoking’s effect on DNA methylation-based indicators of accelerated aging. For example, it is likely that some of the probes used to capture chronological aging in the first-generation DNA methylation-based aging measures will be found to be related to smoking. Third, it is likely that the patterns of association identified in the current study could be generalized to other ethnic or racial groups in addition to African Americans, but that will need to be demonstrated using other samples.

In sum, currently available DNA methylation-based indicators of accelerated aging provide a range of alternative ways to characterize accelerated aging. It is increasingly apparent that they are not simply alternative measures of a unitary underlying construct. In the current study we found very different patterns of association with smoking across the different indices of accelerated aging. This variability may be useful in teasing apart underlying processes that contribute to morbidity and premature death. We also found that DNA methylation-based indicators of smoking capture similar patterns of association as does self-reported smoking, but does so more strongly, typically accounting for, and fully mediating, any observed effects of self-reported smoking. When DNA methylation-based indices of smoking are substituted for self-reported assessments of smoking, they will typically fully capture the impact of self-reported smoking on outcomes. Of additional interest, methylation-based assessment of PACKYRS and cg05575921 typically performed similarly in their association with accelerated aging outcomes and in mediating effects of self-reported smoking on outcomes, with methylation-based pack-years typically performing somewhat better than cg05575921.

## Figures and Tables

**Table 1:** Partial correlation between cigarette use and DNA methylation-based aging indices, controlling for BMI, gender, and cell-types (Monocytes, natural killer, CD8+T, CD4+T, and Bcells) (N = 500)

	Self-Reported Cigarette Consumption		PACKYRS		cg05575921	
	*r*	*p*-Value	*r*	*p*-Value	*r*	*p*-Value
Hannum	0.046	0.304	0.057	0.208	−0.008	0.851
Horvath	−0.007	0.876	−0.056	0.211	0.055	0.225
PhenoAge	0.122 **	0.007	0.133 **	0.003	−0.109 *	0.015
mTL	−0.241 **	6.211 × 10 ^−8^	−0.261 **	3.917 × 10 ^−9^	0.280 **	2.589 × 10 ^−10^
GrimAge	0.650 **	1.409 × 10 ^−60^	0.862 **	1.091 × 10 ^−146^	−0.778 **	5.480 × 10 ^−101^
ADM	0.097 *	0.032	0.163 **	2.910 × 10 ^−4^	−0.148 **	0.001
BM2	0.052	0.253	0.007	0.875	−0.031	0.496
CystatinC	0.099 *	0.028	0.149 **	0.001	−0.085 ^†^	0.059
GDF15	0.222 **	6.319 × 10 ^−7^	0.291 **	4.779 × 10 ^−11^	−0.265 **	2.219 × 10 ^−9^
Leptin	0.001	0.997	0.006	0.901	0.033	0.460
PAI1	0.087 ^†^	0.052	0.080 ^†^	0.077	−0.055	0.220
TIMP1	0.085 ^†^	0.060	0.063	0.162	−0.046	0.307

^†^*p* ≤ 0.10; * *p* ≤ 0.05; ** *p* ≤ 0.01 (two-tailed tests). *Note*: the measure of accelerated aging using the residual scores from the regression of DNA methylation-based age on chronological age; PACKYRS = DNAm-based estimate of smoking pack-years; Hannum = Hannum method; Horvath = Horvath method; PhenoAge = phenotypic aging; mTL = DNA methylation-based telomere length; GrimAge = DNA methylation-based biomarker of mortality risk age; ADM = adrenomedullin; BM2 = beta-2 microglobulin; CystatinC = Cystatin C; GDF15 = growth differentiation factor 15; Leptin = leptin; PAI1 = plasminogen activation inhibitor 1; TIMP1 = tissue inhibitor metalloproteinase 1.

**Table 2:** Regression models depicting the effects of cigarette use on DNA methylation-based aging indices through DNAm-based estimates of smoking, controlling for BMI, gender, and cell-types (Monocytes, natural killer, CD8+T, CD4+T, and Bcells) (N = 500).

		Self-Reported Cigarette Consumption		PACKYRS		cg05575921	
		β	*p*-Value	β	*p*-Value	β	*p*-Value
PhenoAge	Model 1	0.111 **	0.007				
	Model 1A	0.048	0.434	0.089	0.159		
	Model 1B	0.085	0.187			−0.034	0.603
mTL	Model 2	−0.231 **	6.211 × 10 ^−8^				
	Model 2A	−0.092	0.108	−0.168 **	0.005		
	Model 2B	−0.053	0.378			0.211 **	0.001
GrimAge	Model 3	0.596 **	1.409 × 10 ^−60^				
	Model 3A	0.022	0.489	0.801 **	5.826 × 10 ^−88^		
	Model 3B	0.110 **	0.007			−0.644 **	9.494 × 10 ^−44^
ADM	Model 4	0.078 *	0.032				
	Model 4A	−0.043	0.425	0.168 **	0.003		
	Model 4B	−0.035	0.537			−0.149 *	0.011
GDF15	Model 5	0.224 **	6.319 × 10 ^−7^				
	Model 5A	0.014	0.825	0.292 **	1.700 × 10 ^−5^		
	Model 5B	0.042	0.543			−0.241 **	0.001

* *p* ≤ 0.05; ** *p* ≤ 0.01 (two-tailed tests). *Note*: the measure of accelerated aging using the residual scores from the regression of DNA methylation-based age on chronological age; PACKYRS = DNAm-based estimate of smoking pack-years; mTL = DNA methylation-based telomere length; GrimAge = DNA methylation-based biomarker of mortality risk age; ADM = adrenomedullin; GDF15 = growth differentiation factor 15.

**Table 3:** Tests of direct and indirect effects of self-reported cigarette consumption (past 8 years) on DNA methylation-based aging indices through DNA methylation-based estimate of smoking pack-years and cg05575921, controlling age; BMI, gender, and cell-types (*N* = 500).

Predictor	Mediators	Outcomes	Direct Effect	95% CI	Indirect Effect	95% CI	Mediation (%)
Self-reported smoking	PACKYRS	PhenoAge	0.048	(−0.097, 0.163)	0.066	(−0.038, 0.187)	--
	cg05575921		0.085	(−0.050, 0.216)	0.027	(−0.090, 0.127)	--
Self-reported smoking	PACKYRS	mTL	−0.093	(−0.217, 0.031)	−0.126 *	(−0.224, −0.023)	57.534
	cg05575921		−0.054	(−0.173, 0.070)	−0.166 *	(−0.264, −0.065)	75.455
Self-reported smoking	PACKYRS	GrimAge	0.022	(−0.040, 0.083)	0.611 **	(0.548, 0.668)	96.524
	cg05575921		0.113 **	(0.031, 0.192)	0.514 **	(0.448, 0.589)	81.978
Self-reported smoking	PACKYRS	ADM	−0.042	(−0.140, 0.059)	0.124 *	(0.043, 0.197)	74.699
	cg05575921		−0.035	(−0.129, 0.071)	0.116 *	(0.040, 0.198)	76.821
Self-reported smoking	PACKYRS	GDF15	0.014	(−0.098, 0.141)	0.218 **	(0.106, 0.307)	93.966
	cg05575921		0.042	(−0.087, 0.170)	0.188 **	(0.071, 0.287)	81.739

* *p* ≤ 0.05; ** *p* ≤ 0.01 (two-tailed tests). *Note*: the measure of accelerated aging using the residual scores from the regression of DNA methylation-based age on chronological age; BMI, gender, and cell-types (Monocytes, natural killer, CD8+T, CD4+T, and Bcells) were controlled; PACKYRS = DNAm-based estimate of smoking pack-years; mTL = DNA methylation-based telomere length; GrimAge = DNA methylation-based biomarker of mortality risk age; ADM = adrenomedullin; GDF15 = growth differentiation factor 15.

**Table 4:** Test of direct and indirect effects of self-reported cigarette consumption (past 8 years) on epigenetic aging through DNA methylation-based estimate of smoking pack-years and cg05575921 controlling for age; BMI, and gender but not cell-type composition (N = 500).

Predictor	Mediators	Outcomes	Direct Effect	95% CI	Indirect Effect	95% CI	Mediation (%)
Self-reported smoking	PACKYRS cg05575921	PhenoAge	−0.071	(−0.233, 0.075)	0.144 *	(0.011, 0.281)	66.666
			−0.028	(−0.190, 0.109)	0.098	(−0.021, 0.228)	--
Self-reported smoking	PACKYRS cg05575921	mTL	−0.010	(−0.173, 0.135)	−0.161 *	(−0.287, −0.033)	94.152
			0.011	(−0.130, 0.129)	−0.182	(−0.293, −0.087)	94.301
Self-reported smoking	PACKYRS cg05575921	GrimAge	−0.034	(−0.122, 0.040)	0.635 **	(0.563, 0.712)	94.918
			0.038	(−0.066, 0.133)	0.557 **	(0.480, 0.642)	93.613
Self-reported smoking	PACKYRS cg05575921	ADM	−0.091	(−0.199, 0.021)	0.158 *	(0.072, 0.238)	63.454
			−0.091	(−0.200, 0.025)	0.157 *	(0.070, 0.258)	63.306
Self-reported smoking	PACKYRS cg05575921	GDF15	−0.029	(−0.147, 0.098)	0.240 **	(0.123, 0.329)	89.219
			−0.004	(−0.136, 0.132)	0.213 **	(0.094, 0.316)	98.157

*p* ≤ 0.10; * *p* ≤ 0.05; ** *p* ≤ 0.01 (two-tailed tests). *Note*: the measure of accelerated aging using the residual scores from the regression of DNA methylation-based age on chronological age; BMI, and gender were controlled; PACKYRS = DNAm-based estimate of smoking pack-years; mTL = DNA methylation-based telomere length; GrimAge = DNAm-based biomarker of mortality risk age; ADM = adrenomedullin; GDF15 = growth differentiation factor 15.

**Table 5:** Test of direct and indirect effects of self-reported cigarette consumption (past 8 years), excluding former smokers who were not also current smokers, on epigenetic aging through DNA methylation-based estimate of smoking pack-years and cg05575921 (N = 470).

Predictor	Mediators	Outcomes	Direct effect	95% CI	Indirect effect	95% CI	Mediation (%)
Self-reported smoking	PACKYRS cg05575921	PhenoAge	0.031	(−0.123, 0.159)	0.087	(−0.028, 0.223)	--
			0.081	(−0.082, 0.218)	0.034	(−0.104, 0.167)	--
Self-reported smoking	PACKYRS cg05575921	mTL	−0.088	(−0.214, 0.043)	−0.137 *	(−0.256, −0.039)	60.889
			−0.055	(−0.196, 0.068)	−0.170 *	(−0.278, −0.066)	75.556
Self-reported smoking	PACKYRS cg05575921	GrimAge	0.029	(−0.040, 0.094)	0.616 **	(0.548, 0.683)	95.504
			0.106 **	(0.019, 0.192)	0.536 **	(0.460, 0.617)	83.489
Self-reported smoking	PACKYRS cg05575921	ADM	−0.046	(−0.169, 0.068)	0.131 *	(0.059, 0.222)	74.011
			−0.045	(−0.164, 0.061)	0.130 *	(0.041, 0.228)	74.286
Self-reported smoking	PACKYRS cg05575921	GDF15	0.015	(−0.115, 0.148)	0.220 **	(0.110, 0.320)	93.617
			0.036	(−0.110, 0.176)	0.197 **	(0.087, 0.310)	84.549

*p* ≤ 0.10; * *p* ≤ 0.05; ** *p* ≤ 0.01 (two-tailed tests). *Note*: the measure of accelerated aging using the residual scores from the regression of DNA methylation-based age on chronological age; BMI, gender, and cell-types (Monocytes, natural killer, CD8+T, CD4+T, and Bcells) were controlled; PACKYRS = DNAm-based estimate of smoking pack-years; mTL = DNA methylation-based telomere length; GrimAge = DNA methylation-based biomarker of mortality risk age; ADM = adrenomedullin; GDF15 = growth differentiation factor 15.

## Supplementary Materials

The following are available online at https://www.mdpi.com/2073-4425/11/3/311/s1, Table S1: Pearson’s correlation between control variables and study variables Table S2: Pearson’s correlation between cigarette use indices and DNA methylation-based aging indices Table S3: Partial correlation between cigarette use and DNA methylation-based aging indices, controlling for BMI, gender, and cell-types (Monocytes, natural killer, CD8+T, CD4+T, and Bcells) after removing anyone with cotinine greater than 10 who reported no smoking Table S4: Data set used for all analyses including DNA methylation scale scores, self-report data, and demographic variables.
