# Supplementary material for: The Effect of Tobacco Smoking Differs across Indices of DNA Methylation-Based Aging in an African American Sample: DNA Methylation-Based Indices of Smoking Capture These Effects
Source: Genes (Basel). 2020 Mar 14;11(3):311. doi: 10.3390/genes11030311 (PMC7140795; doi:10.3390/genes11030311)
Supplement: Supplementary file 1 [file genes-11-00311-s001.zip › Supplements/Table S1.docx]

**Table S1.** Pearson’s correlation between control variables and study variables (N = 500)

|  | BMI | Gender | Mono | NK | CD8T | CD4T | Bcell |
| --- | --- | --- | --- | --- | --- | --- | --- |
| Hannum | .012 | .180** | .207** | .208** | -.266** | -.409** | .087† |
| Horvath | .025 | .108* | .089* | .088* | .102* | -.122** | -.145** |
| PhenoAge | .095* | -.022 | .240** | .059 | -.225** | -.414** | -.035 |
| mTL | .092* | -.241** | -.215** | -.364** | -.091* | .359** | -.031 |
| GrimAge | -.072 | .312** | .229** | -.057 | -.143** | -.271** | -.083† |
| ADM | .166** | -.534** | .113* | -.171** | -.321** | -.128** | -.073 |
| BM2 | .072 | -.076† | .079† | -.048 | -.144** | -.223** | .040 |
| CystatinC | .087† | .023 | .312** | .020 | -.183** | -.392** | -.170** |
| GDF15 | -.039 | -.008 | .060 | .039 | -.048 | -.180** | -.018 |
| Leptin | .183** | -.697** | -.111* | -.056 | -.087† | .052 | .087† |
| PAI1 | .122** | .277** | .212** | -.012 | -.059 | -.152** | -.362** |
| TIMP1 | .145** | .119** | .256** | -.052 | -.247** | -.333** | -.264** |
| Smoking | -.139** | .157** | -.016 | -.040 | .046 | .079† | .032 |
| PACKYRS | -.194** | .263** | .054 | -.046 | .014 | -.056 | .084† |
| cg05575921 | .190** | -.251** | -.039 | .049 | -.018 | .026 | -.032 |

^†^ *p* ≤ .10; * *p* ≤ .05; ** *p* ≤ .01 (two-tailed tests).

*Note*: Hannum = Hannum method; Horvath = Horvath method; PhenoAge = phenotypic aging; mTL = methylation-based telomere length; GrimAge = DNAm-based biomarker of mortality risk age; ADM = adrenomedullin; BM2 = beta-2 microglobulin; CystatinC = Cystatin C; GDF15 = growth differentiation factor 15; Leptin = leptin; PAI1 = plasminogen activation inhibitor 1; TIMP1 = tissue inhibitor metalloproteinase 1; PACKYRS = DNAm-based estimate of smoking pack-years;
