# Supplementary material for: The Effect of Tobacco Smoking Differs across Indices of DNA Methylation-Based Aging in an African American Sample: DNA Methylation-Based Indices of Smoking Capture These Effects
Source: Genes (Basel). 2020 Mar 14;11(3):311. doi: 10.3390/genes11030311 (PMC7140795; doi:10.3390/genes11030311)
Supplement: Supplementary file 1 [file genes-11-00311-s001.zip › Supplements/Table S2.docx]

**Table S2.** Pearson’s correlation between cigarette use indices and methylomic aging indices (*N* = 500)

|  | Self-reported  cigarette consumption  (past 8 years) | | PACKYRS | | cg05575921 | |
| --- | --- | --- | --- | --- | --- | --- |
|  | *r* | *p*-value | *r* | *p*-value | *r* | *p*-value |
| Hannum | .014 | .750 | .097* | .030 | -.039 | .385 |
| Horvath | -.010 | .828 | -.044 | .331 | .040 | .377 |
| PhenoAge | .049 | .272 | .106* | .018 | -.074† | .096 |
| mTL | -.201** | 6.000E-6 | -.273** | 5.125E-10 | .273** | 5.528E-10 |
| GrimAge | .600** | 2.728E-50 | .830** | 1.237E-128 | -.757** | 4.158E-94 |
|  |  |  |  |  |  |  |
| ADM | -.035 | .440 | -.021 | .633 | .027 | .545 |
| BM2 | .011 | .798 | -.003 | .949 | -.010 | .822 |
| CystatinC | .040 | .368 | .124** | .005 | -.066 | .139 |
| GDF15 | .197** | 9.000E-6 | .277** | 2.833E-10 | -.252** | 1.182E-8 |
| Leptin | -.121** | .007 | -.192** | 1.600E-5 | .214** | 1.000E-6 |
| PAI1 | .081† | .072 | .093* | .037 | -.086† | .055 |
| TIMP1 | .038 | .402 | .059 | .187 | -.048 | .284 |

^†^ *p* ≤ .10; * *p* ≤ .05; ** *p* ≤ .01 (two-tailed tests).

*Note*: the measure of accelerated aging using the residual scores from the regression of methylomic age on chronological age; PACKYRS = DNAm-based estimate of smoking pack-years; Hannum = Hannum method; Horvath = Horvath method; PhenoAge = phenotypic aging; mTL = methylation-based telomere length; GrimAge = DNAm-based biomarker of mortality risk age; ADM = adrenomedullin; BM2 = beta-2 microglobulin; CystatinC = Cystatin C; GDF15 = growth differentiation factor 15; Leptin = leptin; PAI1 = plasminogen activation inhibitor 1; TIMP1 = tissue inhibitor metalloproteinase 1.
