# Supplementary material for: The Effect of Tobacco Smoking Differs across Indices of DNA Methylation-Based Aging in an African American Sample: DNA Methylation-Based Indices of Smoking Capture These Effects
Source: Genes (Basel). 2020 Mar 14;11(3):311. doi: 10.3390/genes11030311 (PMC7140795; doi:10.3390/genes11030311)
Supplement: Supplementary file 1 [file genes-11-00311-s001.zip › Supplements/Table S3.docx]

**Table S3.** Partial correlation between cigarette use and methylomic aging indices, controlling for BMI, gender, and cell-types (Monocytes, natural killer, CD8+T, CD4+T, and Bcells) after removing anyone with cotinine > 10 who reported no smoking (*N* = 469)

|  | Self-reported  cigarette consumption | | PACKYRS | | cg05575921 | |
| --- | --- | --- | --- | --- | --- | --- |
|  | *r* | *p*-value | *r* | *p*-value | *r* | *p*-value |
| Hannum | .051 | .270 | .051 | .276 | .002 | .973 |
| Horvath | -.001 | .979 | -.067 | .153 | .070 | .135 |
| PhenoAge | .125** | .007 | .136** | .003 | -.105* | .024 |
| mTL | -.251** | 4.834E-8 | -.264** | 8.134E-9 | .276** | 1.636E-9 |
| GrimAge | .666** | 1.671E-60 | .864** | 2.912E-139 | -.780** | 1.641E-95 |
|  |  |  |  |  |  |  |
| ADM | .109* | .019 | .168** | 2.850E-4 | -.148** | .001 |
| BM2 | .044 | .343 | .003 | .952 | -.023 | .622 |
| CystatinC | .095* | .041 | .154** | .001 | -.094* | .044 |
| GDF15 | .227** | 8.007E-7 | .287** | 3.341E-10 | -.257** | 2.080E-8 |
| Leptin | -.003 | .955 | .005 | .918 | .030 | .519 |
| PAI1 | .087† | .063 | .074 | .110 | -.047 | .314 |
| TIMP1 | .082† | .080 | .066 | .159 | -.051 | .272 |

^†^ *p* ≤ .10; * *p* ≤ .05; ** *p* ≤ .01 (two-tailed tests).

*Note*: the measure of accelerated aging using the residual scores from the regression of methylomic age on chronological age; PACKYRS = DNAm-based estimate of smoking pack-years; Hannum = Hannum method; Horvath = Horvath method; PhenoAge = phenotypic aging; mTL = methylation-based telomere length; GrimAge = DNAm-based biomarker of mortality risk age; ADM = adrenomedullin; BM2 = beta-2 microglobulin; CystatinC = Cystatin C; GDF15 = growth differentiation factor 15; Leptin = leptin; PAI1 = plasminogen activation inhibitor 1; TIMP1 = tissue inhibitor metalloproteinase 1.
